# Ocular Drug Delivery to the Retina: Current Innovations and Future Perspectives

**DOI:** 10.3390/pharmaceutics13010108

**Published:** 2021-01-15

**Authors:** Hyeong Min Kim, Se Joon Woo

**Affiliations:** Department of Ophthalmology, Seoul National University College of Medicine, Seoul National University Bundang Hospital, Seongnam 13620, Korea; hmkim3@gmail.com

**Keywords:** intravitreal injection, ocular drug delivery, nanoparticle, implant, hydrogel

## Abstract

Treatment options for retinal diseases, such as neovascular age-related macular degeneration, diabetic retinopathy, and retinal vascular disorders, have markedly expanded following the development of anti-vascular endothelial growth factor intravitreal injection methods. However, because intravitreal treatment requires monthly or bimonthly repeat injections to achieve optimal efficacy, recent investigations have focused on extended drug delivery systems to lengthen the treatment intervals in the long term. Dose escalation and increasing molecular weight of drugs, intravitreal implants and nanoparticles, hydrogels, combined systems, and port delivery systems are presently under preclinical and clinical investigations. In addition, less invasive techniques rather than intravitreal administration routes, such as topical, subconjunctival, suprachoroidal, subretinal, and trans-scleral, have been evaluated to reduce the treatment burden. Despite the latest advancements in the field of ophthalmic pharmacology, enhancing drug efficacy with high ocular bioavailability while avoiding systemic and local adverse effects is quite challenging. Consequently, despite the performance of numerous in vitro studies, only a few techniques have translated to clinical trials. This review discusses the recent developments in ocular drug delivery to the retina, the pharmacokinetics of intravitreal drugs, efforts to extend drug efficacy in the intraocular space, minimally invasive techniques for drug delivery to the retina, and future perspectives in this field.

## 1. Introduction

Retinal diseases, such as neovascular age-related macular degeneration (AMD), diabetic retinopathy, and retinal vascular disorders, are the leading causes of vision deterioration in most developed countries [1]. The recent development of anti-vascular endothelial growth factor (anti-VEGF) treatments has markedly suppressed disease progression [2,3,4,5,6]. Current anti-VEGF drugs, including bevacizumab (Avastin; Genentech, Inc., San Francisco, CA, USA), ranibizumab (Lucentis; Genentech, Inc., San Francisco, CA, USA), and aflibercept (Eylea; Regeneron, Inc., Tarrytown, NY; and Bayer Healthcare Pharmaceuticals, Berlin, Germany) are manufactured as humanized monoclonal antibodies. However, according to their intraocular pharmacokinetic properties, these biologic drugs have relatively short half-lives, thereby requiring monthly or bi-monthly injections to maintain their efficacy in the intraocular space [7,8,9,10]. Although anti-VEGF treatment is effective and beneficial for numerous retinal disorders, frequent intravitreal drug injections become a significant treatment burden to patients and the healthcare system owing to the overall cost and invasive technique utilized [11,12,13,14].

**Figure 1:**
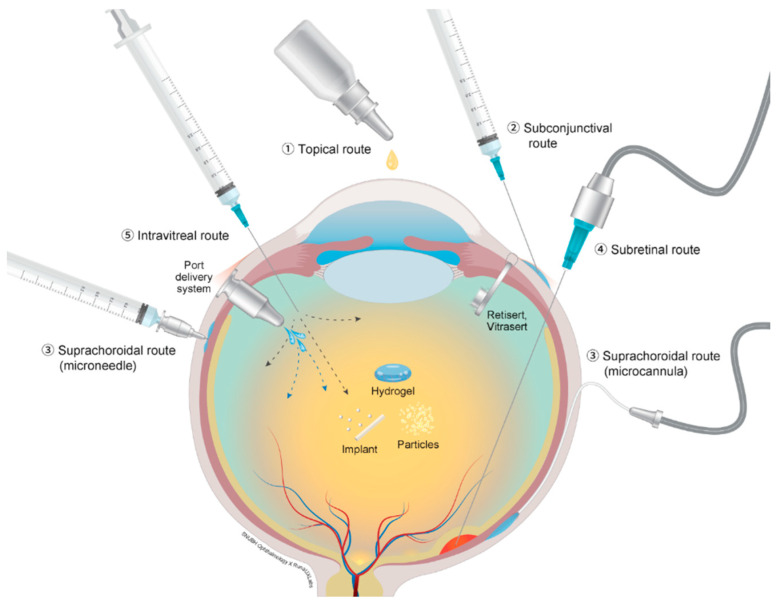
Schematic of several ocular drug administration routes: (1) topical route, (2) subconjunctival route, (3) suprachoroidal route with microcannula and microneedle, (4) subretinal route, and (5) intravitreal injection and port delivery system.

Several advancements in ocular drug delivery systems for achieving intraocular drug efficacy have been observed. Based on the intraocular drug dose escalation performed in the HARBOR and SAVE clinical trials for neovascular AMD, limiting intravitreal injections has been proposed to relieve the socioeconomic treatment burden [15,16,17,18]. Recently, with the help of tissue engineering, hydrogels, micro- and nanoparticles, and several other intravitreally administered drug delivery systems have been investigated. These surface-conjugate-modified drug delivery systems can enhance delivery efficiency by prolonging intravitreal half-lives due to the sustained release of the drug, better biocompatibility, and protection from biological drug degradation [19,20,21,22]. Several periocular drug administration routes, which are considered less invasive than intravitreal injections, have been investigated [23,24,25]. Figure 1 presents several ocular drug delivery routes, including topical, subconjunctival, suprachoroidal, subretinal, and trans-scleral, and intravitreal. In this review, we aim to summarize (1) intraocular pharmacokinetics of current intravitreal drugs; (2) efforts to enhance the intraocular pharmacokinetics and pharmacodynamics of intravitreal drugs: dose escalation of intravitreal drugs, increasing the molecular weight of intravitreal drug molecules, sustained-release Intravitreal implants, micro- and nanoparticles, hydrogels, combined drug delivery systems, port delivery systems; and (3) Drug administration routes besides the intravitreal route.

## 2. Intraocular Pharmacokinetics of Current Intravitreal Drugs

The intravitreal drugs that are currently administered globally for retinal diseases are ranibizumab, bevacizumab, aflibercept, brolucizumab, faricimab, and conbercept. These VEGF inhibitors are produced as recombinant humanized monoclonal antibodies, such as ranibizumab (antigen-binding fragments, Fab, molecular weight 49 kDa) and bevacizumab (IgG, molecular weight 148 kDa), and fusion proteins such as aflibercept (Fc, molecular weight 145 kDa). Previous studies have investigated the intraocular pharmacokinetics of anti-VEGF drugs to achieve maximal treatment efficacy [8,9,10,26,27,28,29,30,31,32,33,34]. In fact, Bakri et al. experimented with Dutch-belted rabbits and reported that the vitreous half-life of 0.5 mg intravitreal ranibizumab was 2.88 days, and that of 1.25 mg bevacizumab was 4.32 days [8,9]. Park et al. experimented with New Zealand white rabbits and reported that the vitreous half-life of ranibizumab was 2.18 days; bevacizumab, 7.56 days; and aflibercept, 3.92 days [32,34]. Kim et al. suggested that intravitreally administered drugs are rapidly distributed in the aqueous humor and retina/choroid for clearance and remain in these tissues for longer periods [35]. Ahn et al. analyzed the intraocular pharmacokinetics of ranibizumab in vitrectomized and non-vitrectomized rabbit eyes. As a result, they reported that the vitreous half-life was 2.51 days in vitrectomized eyes and 2.75 days in non-vitrectomized eyes. However, the overall vitreous clearance rate and mean concentrations did not reveal any statistically significant differences between vitrectomized and non-vitrectomized eyes [28,30]. Niwa et al. measured the pharmacokinetic parameters in vitrectomized and non-vitrectomized monkey eyes; the respective aqueous half-lives of ranibizumab were 1.4 days and 2.3 days while those of aflibercept were 1.5 days and 2.2 days [31]. Based on this animal study, the half-life of ranibizumab and aflibercept was shorter in vitrectomized eyes than in non-vitrectomized eyes. The latest anti-VEGF agent, brolucizumab, is currently commercially available. According to the FDA nonclinical report, the vitreous half-life of brolucizumab in a single-dose New Zealand white rabbit study and cynomolgus monkeys was 2.94 days and 2.08 days, respectively [36]. In a human clinical trial (RTH258-E003), the vitreous half-life was 5 ± 2 days after intravitreal injection of 6 mg brolucizumab [36]. Another anti-VEGF drug, faricimab, has also been newly developed and is currently in clinical trial. Regula et al. reported a half-life of 2.83 days in a single-dose study in cynomolgus monkeys [37]. Conbercept, a fusion protein with decoy VEGF receptors, was also introduced, and its intravitreal half-life was 4.24 days in a single-dose study with Chinchilla rabbits [38].

In addition to pre-clinical animal studies, pharmacokinetic parameters in human eyes have also been measured. Krohne et al. calculated the intraocular pharmacokinetics after a single intravitreal injection of bevacizumab and ranibizumab [26,27]. The aqueous half-life of bevacizumab was 9.82 days while that of ranibizumab was 7.19 days in human non-vitrectomized eyes. Both drug concentrations peaked on the first day after injection to the aqueous humor and declined monoexponentially. Previous studies on intravitreal pharmacokinetics in animal and human eyes are summarized in Table 1.

Current anti-VEGF drugs are rather heavy molecules with low clearance rate compared to small molecules. Moreover, good bioavailability in the vitreous cavity and gradual distribution after intravitreal injection make these drugs appealing. However, relatively short intravitreal half-lives are still not satisfactory for both clinicians and patients, leading to the treatment burden. Metabolic drug elimination such as matrix metalloproteinases, trypsin, esterase, peptidase, and other enzymes might be involved in the vitreous [39], however, it is not well explored and thus considered less important. Therefore, both anterior and posterior clearance pathway influence intravitreal half-lives significantly.

For the intraocular pharmacokinetics and pharmacodynamics of anti-VEGF drugs, Hutton-Smith et al. suggested a mechanistic model that predicts intravitreal half-lives according to the molecular weight of VEGF-binding molecules, dose, and dissociation constant (K_D_) [40]. After intravitreal injection, anti-VEGF molecules are distributed to the posterior space and penetrate the inner limiting membrane (ILM) and retinal pigment epithelium (RPE) to affect the retina/choroid. Elimination occurs through the anterior chamber and retina. As a result, Hutton-Smith et al. proposed a three-compartment model (aqueous, vitreous, and retina) that simulates the impact of ILM and RPE barriers as well as aqueous humor and retinal elimination outflow [41,42]. Saunders et al. focused on patients with neovascular AMD that were administered ranibizumab; the elimination half-life of vascular endothelial growth factor A (VEGF-A) in the aqueous humor was 3.5 days, and the duration of complete VEGF-A suppression was 41 days [43]. With reference to previously described models, dose escalation, sustained release of implants or particles, protection of degradation, and high ILM and RPE permeability, established by molecular engineering of anti-VEGF drugs, could prolong the duration of VEGF suppression.

**Table 1:** Intraocular pharmacokinetics of current and developing anti-VEGF drugs for animal and human eyes.

Drug	Species	Molecular Weight (kDa)	Intravitreal t_1/2_ (day)	Reference
Pegaptanib	Rhesus monkey	40	3.92	Drolet et al. [44]
Ranibizumab	New Zealand rabbit	48	2.51 (vitrectomized)	Ahn et al. [30]
			2.75 (nonvitrectomized)	
			2.18	Park et al. [34]
			3.0	Gaudreault et al. [10]
			3.2	Shatz et al. [45]
	Dutch belted rabbit		2.88 2.81	Bakri et al. [8] Christoforidis et al. [46]
	Cynomolgus monkey		1.4 (vitrectomized)	Niwa et al. [31]
			2.3 (nonvitrectomized)	
			2.54	Gaudreault et al. [47]
			2.63	
	Owl monkey		2.73	Christoforidis et al. [48]
	Human		7.19 (nonvitrectomized)	Krohne et al. [27]
Bevacizumab	New Zealand rabbit	149	7.56	Ahn et al. [33]
			6.51	Sinapis et al. [49]
	Dutch belted rabbit		4.32	Bakri et al. [9]
			6.0	Nomoto et al. [50]
			4.22	Christoforidis et al. [46]
	Owl monkey		3.60	Christoforidis et al. [48]
	Human		9.82 (nonvitrectomized)	Krohne et al. [26]
			11.67	Meyer et al. [51]
Aflibercept	New Zealand rabbit	145	3.92	Park et al. [34]
	Cynomolgus monkey		1.5 (vitrectomized) 2.2 (nonvitrectomized)	Niwa et al. [31]
	Owl monkey		2.44	Christoforidis et al. [48]
Abicipar pegol	Human	34	≥13 days	Campochiaro et al. [52]
Brolucizumab	New Zealand rabbit	26	2.94	FDA review [36]
	Cynomolgus monkey		2.08	
	Human		5 ± 2	
Faricimab	Cynomolgus monkey	150	2.83 (aqueous)	Regula et al. [37]
Conbercept	Chinchilla rabbit	143	4.24	Li et al. [38]

## 3. Efforts to Enhance the Intraocular Pharmacokinetics and Pharmacodynamics of Intravitreal Drugs

This section presents previous, current, and future investigations that aim to enhance the intraocular pharmacokinetics and pharmacodynamics of intravitreal drugs. From dose escalation to intravitreal implants and tissue engineered nanoparticles, cutting-edge techniques and advancements have been accomplished.

### 3.1. Dose Escalation of Intravitreal Drugs

Two hallmark clinical trials, HARBOR and SAVE, have investigated the dose escalation of ranibizumab for neovascular AMD. Both studies compared the effects of 2 mg vs. 0.5 mg (conventional) of ranibizumab administered monthly and/or PRN (as needed) regimens. In the HARBOR study, longer intravitreal injection intervals (12.5 weeks in the 2 mg PRN group vs. 9.9 weeks in the 0.5 mg PRN group) were found, and comparable visual acuity improvements were achieved (+10.1 letters in the 2 mg PRN group vs. +8.2 letters in the 0.5 mg PRN group) [18]. In the SAVE study, recalcitrant patients administered standard doses and regimens were included and injected with 2 mg doses of ranibizumab [15]. The results also showed better visual acuity improvements than those in the standard treatment guidelines. These findings suggest that the dose escalation of anti-VEGF drugs for neovascular AMD enables longer and deeper retinal VEGF suppression, consistent with the three-compartment model described by Hutton-Smith et al. [41,42].

Other subsequent studies with a focus on administering higher doses have been performed since the HARBOR and SAVE trials. Chan et al. proposed that the 2-mg dose of ranibizumab resulted in more rapid improvements than the 0.5 mg dose; however, the primary endpoint at 12 months did not show significant differences in visual outcomes [53]. The READ-3 study group that evaluated diabetic macular edema suggested that conventional 0.5 mg and higher 2 mg doses of ranibizumab did not alter the 24 months visual improvements [54]. In a pre-clinical animal study, Kim et al. administered a 10-fold dose of ranibizumab in rabbit eyes that resulted in a two-fold increase in retinal half-life and long-lasting effective concentration in the retinal compartment, without any adverse local or systemic effects [55]. The contradictory results of dose escalation suggest that further experiments and clinical trials should be conducted. Currently, randomized, double-masked, phase 3 clinical trials are being conducted with high-dose aflibercept every 12 or 16 weeks after a loading phase in participants with diabetic retinopathy (PHOTON; NCT04429503) and neovascular AMD (PULSAR; NCT04423718).

Recently, the latest anti-VEGF drug comprising of single-chain antibody fragments, brolucizumab, entered the market. Small molecular weight (26 kDa), higher tissue penetration, and more condensed concentration than current anti-VEGF drugs allowed clinicians to administer fewer injections (a 12 week treatment interval) [56,57]. The HAWK (brolucizumab 3 mg, 6 mg, or aflibercept 2 mg) and HARRIER (brolucizumab 6 mg or aflibercept 2 mg) clinical trials were designed to compare brolucizumab to aflibercept for neovascular AMD [58]. After consecutive loading phases at weeks 0, 4, and 8, brolucizumab was intravitreally injected at 8 or 12 week intervals. The visual functional outcomes at the primary end-point at week 48 resulted in no differences between brolucizumab and aflibercept (HAWK: +6.6 (6 mg) and +6.1 (3 mg) letters vs. +6.8 letters; HARRIER: +6.9 (6 mg) letters vs. +7.6 letters), while the anatomical improvements (central subfield thickness reductions) of brolucizumab were superior to aflibercept (HAWK: −172.8 μm vs. −143.7 μm; HARRIER: −193.8 μm vs. −143.9 μm). Consequently, anti-VEGF drugs have been designed as monthly (ranibizumab) or bi-monthly (aflibercept) for longer interval (brolucizumab) regimens, ultimately reducing treatment nonadherence, burden, and patient dissatisfaction.

### 3.2. Increasing the Molecular Weight of Intravitreal Drug Molecules

Contrary to recent development of small molecules, such as brolucizumab (26 kDa) and abicipar pegol (34 kDa), KODIAK sciences (Palo Alto, CA, USA) have developed KSI-301 (950 kDa), an anti-VEGF monoclonal antibody conjugated with a large polymer to increase its molecular weight. Larger molecules are known to enhance intraocular stability and prolong intravitreal half-life [59] in terms of slow diffusion in the vitreous cavity and decreased elimination process of both anterior and posterior pathway, affecting permeability across the retinal pigment epithelium (RPE), inner limiting membrane, and blood-retina barrier. As a result, novel therapeutics are currently in phase 2/3 human clinical trials (DAZZLE, NCT04049266) [60]. A previous phase 1 study (NCT03790852) resulted in visual improvements, with 6 months interval intravitreal injections in patients with neovascular AMD, diabetic macular edema, and retinal vein occlusion [61].

### 3.3. Sustained-Release Intravitreal Implants

Some intravitreal implants, Ozurdex^®^ (Allergan Inc., Irvine, CA, USA), Retisert^®^ (Bausch & Lomb, Rochester, NY, USA), and Illuvien^®^ (Alimera Sciences Inc., Alpharetta, GA, USA), have been approved by the FDA for diabetic macular edema, macular edema secondary to retinal vascular occlusion, and posterior uveitis. Ozurdex^®^ (dexamethasone) and Retisert^®^/Illuvien^®^ (fluocinolone acetonide) intravitreal implants target the retinal layer, with higher drug dose and longer drug concentration in the intraocular space [62,63,64]. Intravitreal implants are categorized as biodegradable and non-biodegradable.

Ozurdex^®^ is a representative biodegradable intravitreal implant with poly (lactic-co-glycolic acid) conjugation. Biodegradable implants exhibit drug efficacy for 3 to 6 months due to the exponential decrease in drug concentration caused by three-dimensional drug release and gradual degradation of scaffolds in the vitreous cavity. Another biodegradable intravitreal implant, the brimonidine drug delivery system (Brimo DDS^®^, Allergan Inc., Irvine, CA, USA), was employed in a phase 2 study (NCT00658619) for patients with geographic atrophy secondary to neovascular AMD [65]. After treatment with brimonidine implants, the geographic atrophy area was reduced at month 12; the injection interval was 6 months. Two definitive phase 3 studies of the second-generation Brimo DDS^®^ (200 μg and 400 μg doses; IMAGINE and ENVISION) are scheduled to commence soon.

Retisert^®^ and Illuvien^®^ are both non-biodegradable and are formed via polymer conjugation. As non-biodegradable implants last for 2 to 3 years, sustained drug release is achieved by the steady linear decrease in drug concentration in the confined diffusion area. However, the devices remain in the vitreous cavity without decaying. Another non-biodegradable implant, Vitrasert^®^ (Bausch & Lomb, Rochester, NY, USA), is known for the sustained release of ganciclovir in the intraocular space for the treatment of cytomegalovirus (CMV) retinitis.

### 3.4. Micro- and Nanoparticles

**Figure 2:**
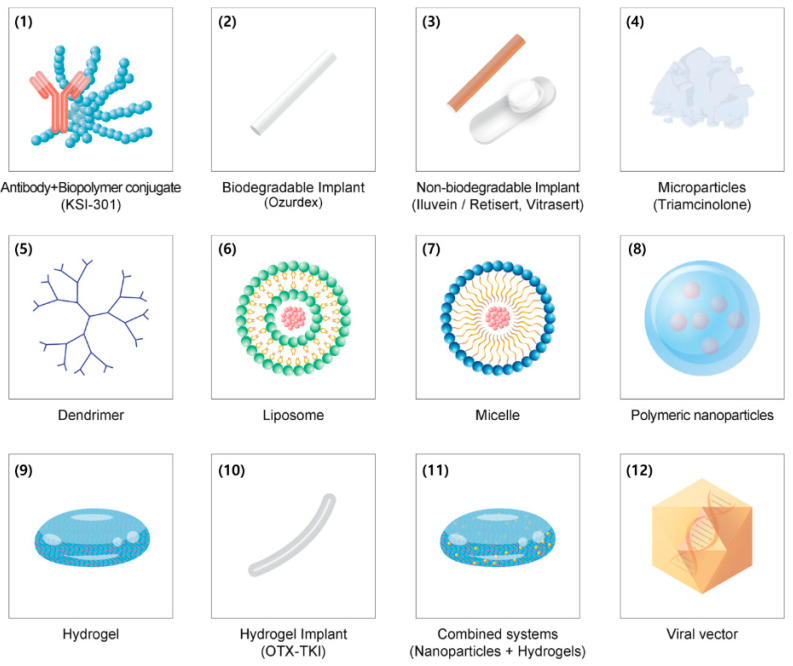
Schematic depicting the latest advancement in multiple ocular drug delivery systems: (**1**) Antibody+Biopolymer conjugate, (**2**) Biodegradable intravitreal implant, (**3**) Non-biodegradable intravitreal implant, (**4**) Triamcinolone acetonide microparticles, (**5**) Dendrimer, (**6**) Liposome, (**7**) Micelle, (**8**) Polymeric nanoparticles, (**9**) Hydrogel, (**10**) Hydrogel implant, (**11**) Combined systems, and (**12**) Viral vector.

Microparticles and nanoparticles have been engineered to deliver drugs efficiently into the intraocular space and can encapsulate different types of molecules. Owing to the modification and fabrication of particle surfaces, compositions, polysaccharide mixtures, and ionic charges, these injected particles are distributed in the vitreous, enabling prolonged intravitreal half-lives by sustained drug release and delayed degradation and elimination [20,21]. From micromolecules to antibodies, nucleic acids, and anti-VEGF drugs, the micro and nanoparticle delivery system has been extensively investigated [19]. Manipulation of nanoparticles with a negative charge is important in the intraocular space as the vitreous matrix is anionic due to collagen and glycosaminoglycan (hyaluronic acid and heparan sulfate) [66]. The size of nanoparticles is also crucial for penetration of the retinal layer, considering the ILM 10–20 nm nanopore meshwork [67,68]. Recently, Kim et al. proposed that large 200 and 250 nm-sized nanoparticles remained longer in the vitreous after intravitreal injection compared to small nanoparticles (25 and 50 nm) due to a blocked posterior elimination pathway [69]. In this section, we describe several types of micro and nanoparticles studied, designed, and tested in previous reports; they are also summarized in Figure 2 and Table 2.

#### 3.4.1. Microparticles

Microparticles have been introduced as potent drugs that can be administered intravitreally for various retinal diseases. Triamcinolone acetonide (TA) has been commercialized as an intravitreal microparticle depot for the treatment of diabetic macular edema, retinal vein occlusion, and posterior uveitis, and is employed to visualize the posterior hyaloid during vitrectomy. Kenalog^®^ (Bristol-Myers Squibb Company, New York, NY, USA), MaQaid^®^ (Wakamoto Pharmaceutical, Tokyo, Japan), Trivaris^®^ (Allergan Inc., Irvine, CA, USA), and Triesence^®^ (Alcon Pharmaceuticals, Ft. Worth, TX, USA) are commercially available and are widely used in ophthalmic practice. Except Kenalog^®^, other TA drugs are preservative-free. According to Chen et al., preservative-free TAs have smaller particle size, with uniform distribution, slower dissolution, and lower free drug level in the vitreous, leading to better and longer duration of drug efficacy than preserved TA [70]. These microcrystal TA drug particles form a depot after intravitreal injection, thereby inducing controlled release and long-lasting therapeutic effects [71]. Missel et al. suggested that the depot formulation decreases the dissolution rates within the vitreous cavity [72]. Previously, intravitreal TA half-life was estimated to be 18.6 days in non-vitrectomized eye [73]. Kim et al. calculated the intravitreal half-life of a depot formulation preservative-free TA compound (24 days) and Kenalog^®^ (23 days) in New Zealand white rabbits, which lasted for as long as 120 days [74].

Recently, a depot formulation of sunitinib malate (GB-102, GrayBug Vision, Redwood City, CA, USA), a tyrosine kinase inhibitor targeting both VEGF-A and platelet-derived growth factor (PDGF), was developed and tested in a human clinical trial. ADAGIO (NCT03249740) and ALTISSIMO (NCT03953079) were phase 2 trials for neovascular AMD. Another phase 2a study (NCT04085341) that included patients with diabetic macular edema and retinal vein occlusion was performed [75]. The ADAGIO study showed that 88% of patients at 3 months and 68% of patients at 6 months were maintained on a single intravitreal injection of GB-102. The ALTISSIMO phase 2b study was subsequently initiated to compare the visual outcomes after intravitreal administration of 1 mg and 2 mg GB-102 and 2 mg aflibercept. Previously, some pre-clinical studies with sunitinib malate suggested its anti-angiogenesis effects in vitro and in vivo as well as the minor ocular toxicity that it induces [75,76,77]. The latest investigation by Tsujinaka et al. proposed that biodegradable polymer sunitinib microparticles self-aggregate into a depot formulation in the vitreous and maintain a steady therapeutic effect in the retinal pigment epithelium/choroid and retina for more than 6 months in an ocular neovascularization minipig model [78].

#### 3.4.2. Dendrimer

Dendrimers are synthetic three-dimensional repeating-unit branched macromolecules; polyamidoamine (PAMAM) and DAB polypropyleneimine (PPI/DAB) are widely used dendrimers for biomedical purposes [79,80]. Dendrimer particles encapsulate drug molecules, enabling controlled release in the intraocular space. Marano et al. developed and investigated a dendrimer-conjugated anti-VEGF agent using a laser-induced choroidal neovascularization (CNV) model. Based on their findings, penetration into the retinal layer and reduction of CNV were achieved for 6 months [80]. Iezzi et al. intravitreally injected dendrimer-based fluocinolone acetonide into the eyes of a retinal degeneration model and observed suppression of microglial neuroinflammation [81]. Kambhampati et al. produced PAMAM dendrimer-conjugated triamcinolone acetonide and observed improved drug efficacy and VEGF suppression [82]. Similarly, Yavuz et al. demonstrated that dexamethasone-conjugated dendrimers were retained longer in the intraocular space than conventional drug delivery [83]. Dabkowska et al. also revealed that the neurotrophin 4-conjugated dendrimer exhibited sustained release of molecules for more than 1 month [84]. Recently, Yang et al. designed cyclic arginine-glycine-aspartate hexapeptide and penetratin-modified dendrimers as drug carriers and reported their potency for maintaining intraocular concentration longer than conventional intravitreal injection [85].

#### 3.4.3. Liposome

Liposomes are synthetic vesicles comprising one or more lipid bilayers and an aqueous core; as a result, they are amphiphilic. Liposomes can encapsulate different types of drug molecules, regardless of their physicochemical properties. Liposomes have been widely used to develop topical therapeutics, such as ciprofloxacin, fluoroquinolone, and fluconazole, that exhibit enhanced corneal and conjunctival permeability [86,87]. Previously, the intravitreal injection of liposomes was investigated for retinal diseases. Compared to the free drug, the intravitreal half-lives of amikacin and clindamycin were found to be extended with the liposomal formulation [88]. Abrishami et al. designed and evaluated nanoliposome-encapsulated bevacizumab. Based on their findings, the retained anti-VEGF drug concentration in the intraocular space was higher than that of free bevacizumab [89]. Besides drug molecules, siRNA encapsulated in the liposome displayed improved intracellular delivery relative to naked siRNA in the CNV model [90]. Overall, liposomes appear to have the potential to be a drug delivery system to the retina and choroid; however, further studies with tissue engineering should be conducted to improve their biocompatibility in the intraocular space.

#### 3.4.4. Polymeric Micelles

Micelles are nanosized amphiphilic core-shell (hydrophobic core, hydrophilic shell) carriers with polymeric surfactants [91,92]. Polyethylene glycol (PEG) and polyethylene oxide (PEO) are commonly used materials for hydrophilic shells while polyglycolic acid (PGA), polycaprolactone (PCL), polylactic acid (PLA), and polylactide/glycolide (PLGA) are widely used materials for hydrophobic cores [93]. These structural designs can increase bioavailability and prolong the residence time of drugs in the intraocular space. Moreover, accumulation in the inflamed area and greater permeability in the retinal layer can be achieved [91]. Micelles are applied for both anterior segment and posterior segment diseases. Numerous studies have focused on retinal neovascularization, retinal degenerative diseases, diabetic retinopathy, and uveitis [91,92,93,94,95,96,97,98]. In fact, Elsaid et al. prepared sirolimus-loaded micelles for a macular degeneration model and found that it increased scleral permeability [94]. Prima et al. developed triamcinolone acetonide-conjugated micelles, which were found to extend drug concentrations in the intraocular space longer than the naked drugs [95]. By using genistein-loaded micelles in a retinal neovascularization model, Li et al. observed significant suppression of neovascularization [98]. Compared to the drug solution itself, micelles achieved better anti-inflammatory effect [96,97]. Because micelles have the advantages of high permeability, low toxicity, and in vivo drug efficacy, further studies should be performed for clinical application.

#### 3.4.5. Polymeric Nanoparticles

Polymeric nanoparticles are produced as nanocapsules or nanospheres that contain drugs that are either encapsulated or dispersed in the polymer matrix. Similar to polymeric micelles, PGA, PLA, and PLGA are mainly used as polymer materials. Because a single polymer molecule can be unstable in the intraocular space due to self-aggregation, stabilizers such as polyvinyl acetate (PVA) and polyvinylpyrrolidone (PVP) are added to the polymer formulation. These stabilizers were employed in the previous literature on techniques such as solvent evaporation, salting-out, and nanoprecipitation, aiding polymeric nanoparticle compounds to distribute stably in the vitreous cavity [98]. Recently, retinal neovascularization-targeted polymeric nanoparticles with anti-VEGF conjugation successfully showed sustained drug release and improved drug efficacy. Ye et al. prepared PLGA nanoparticles loaded with bevacizumab that were administered intravitreally into rabbit eyes; a substantial increase in the following pharmacokinetic parameters was achieved: intravitreal half-lives, mean concentration, and bioavailability compared with those of the free drug [99]. After developing dexamethasone-loaded PLGA/PEI nanoparticles with conjugated bevacizumab for a CNV model, Liu et al. proposed a strong anti-angiogenic effect as demonstrated via in vitro and in vivo studies [100].Qiu et al. designed fenofibrate-loaded PLGA-nanoparticles that were injected intravitreally into the eyes of rats with diabetic retinopathy and a neovascularization model. Accordingly, reduction of retinal vascular leakage, suppression of VEGF and CNV formation, and inhibition of retinal leukocytosis were observed [101].

#### 3.4.6. Solid Lipid Nanoparticles

Solid lipid nanoparticles consist of lipids, such as triglycerides, fatty acids, and surfactants. These nanocarriers can encapsulate micromolecules, siRNA, DNA, and proteins. Abrishami et al. injected solid lipid nanoparticle-conjugated diclofenac intravitreally and found that mild improvement in drug efficacy occurred in the intraocular space [102]. After sunitinib-encapsulated solid lipid nanoparticles were administered intravitreally into rabbit eyes, drug efficacy was enhanced [103].

#### 3.4.7. Coated Nanoparticles

Human serum albumin (HSA), chitosan, and cyclodextrin nanoparticles are also types of nanoparticles. Previously, Huang et al. developed hyaluronic acid-coated HSA nanoparticles and injected them into retinal ischemia-reperfusion injury rat model eyes. Based on their results, the nanoparticles suppressed retinal degeneration [104]. By administering HSA nanoparticles via suprachoroidal injection in retinal degeneration rat model, Tzameret et al. found that the structure and function of the retina were significantly different between treated eyes and control eyes [105]. Kim et al. evaluated brimonidine-loaded HSA nanoparticles in optic nerve crush rat model. Based on their findings, these nanoparticles reduced Aβ-induced retinal ganglion cell (RGC) death, suggesting a neuroprotective effect [106]. By assessing bevacizumab-loaded HSA nanoparticles, Redin et al. noticed that after an initial burst, continuous release of bevacizumab was observed, with no definite RPE cell toxicity [107]. Varshochian et al. also developed HSA-PLGA-nanoparticles loaded with bevacizumab and administered the nanoparticles via intravitreal injection to determine their effect on neovascularization suppression [108]. According to their results, vitreous bevacizumab concentrations were retained for 8 weeks in rabbit eyes. Chitosan-based microparticles containing bevacizumab were designed by Jiang et al.; they reduced the initial burst release and extended the drug release period up to 6 months [109].

#### 3.4.8. Inorganic Nanoparticles

Inorganic nanoparticles, such as gold, silver, silicone, iron oxide, zinc oxide, cerium oxide, titanium oxide, and magnetic nanoparticles have been investigated to derive their potential for use in drug delivery. Gold nanoparticles are gaining popularity owing to their antioxidant, anti-inflammatory, and antiangiogenic effects [110]. Gold nanoparticles also possess several advantages, such as their relatively small size and high biocompatibility. They also possess the ability to conjugate different drug molecules and easily modify particle surface [111]. Kim et al. applied gold nanoparticles in a retinal neovascularization model and revealed that VEGF-mediated angiogenesis and cell proliferation/migration were significantly inhibited [112]. Recently, Apaolaza et al. designed HA-coated gold nanoparticles to increase mobility in the vitreous and increase stability by CD44 receptor interaction. Accordingly, better suppression of retinal neovascularization was achieved by inhibiting advanced glycation end-products (AGEs) related to RPE cell death, VEGF, interleukins, and reactive oxygen species (ROS) [113]. Cerium oxide and magnetic nanoparticles have also been investigated as possible anti-inflammatory and antiangiogenic agents that target the RPE and choroidal layer [114,115,116]. Silicone quantum dots injected intravitreally in a rat model of retinal degeneration showed beneficial effect in cell survival rate and improving electroretinogram (ERG) patterns [117]. Another experiment with zinc oxide nanoparticles in murine photoreceptor-derived cells suggested suppressed cell proliferation and migration, and reduced production of transforming growth factor (TGF-β) and matrix metalloproteinases (MMP-9) [118].

### 3.5. Hydrogels

Hydrogels are multi-dimensional network structures with chemically or physically cross-linked polymer chains that are biocompatible in the intraocular space [119,120]. Chemical processes occur because of the covalent bonds between polymer chains, whereas physical processes occur because of ionic, hydrogen, hydrophobic, and van der Waals forces [121,122]. Because these bonds allow stability, sustained drug release and maintenance of drug concentration in the vitreous may be possible. For natural polymers, polysaccharides, such as hyaluronic acid (HA), chitosan, and dextran, are widely used to design hydrogels. HA-based hydrogels are negatively charged, and protein degradation and drug release in the vitreous could be controlled by modifying the physicochemical properties of the hydrogel molecule [123]. Chitosan is a cationic nanocarrier that can interact with anionic polymers to form hydrogels. For synthetic polymers, PEG, PVA, PAM, PCL, and PLGA are commonly used to produce hydrogels [124]. A phase 1 randomized, multicenter clinical trial of a tyrosine kinase inhibitor implant conjugated with a hydrogel (Ocular Therapeutix, Bedford, MA, USA) is currently in progress [125]. Tyrosine kinase inhibitors can suppress VEGF-induced retinal vascular leakage. In fact, preclinical data revealed sustained drug delivery for up to 12 months [126].

Recently, in situ hydrogel systems have been developed to efficiently deliver drugs to the posterior segment of the eye. Injectable in situ hydrogels are administered intravitreally, and with external stimuli (temperature, light, pH level, ionic), are converted to a gel form [127,128,129,130,131,132,133,134,135]. Because the sol-gel transition has multiple advantages, including accurate dosing, easy administration, and prolonged intravitreal half-lives and sustained drug release, these systems have been extensively investigated. Thermoresponsive hydrogels are developed in liquid form at room temperature and transformed into gel at body temperature after administration. These hydrogels have also been extensively studied for the delivery of anti-VEGF drugs to the retina. Rauck et al. demonstrated that bevacizumab-loaded hydrogels exhibited sustained drug release for up to 9 weeks in a rabbit model [130]. Yu et al. prepared bevacizumab-loaded vinylsulfone-functionalized HA (HA-VS) and thiolated dextran (Dex-SH) hydrogels; bevacizumab concentration in the intraocular space was found to be maintained for at least 6 months in rabbit eyes with in situ gel formation [131]. By administering ranibizumab and aflibercept-loaded hydrogels, Osswald et al. found that the anti-angiogenic effects were maintained for up to 12 weeks. In addition, VEGF was found to be significantly inhibited in vitro and in vivo [132,133]. Liu et al. reported that in situ gel formation with crosslinking reduced degradation, thereby maintaining drug concentration in vitro for 6 months [135]. Light-activated hydrogels are initiated by the photoinitiator 2-dimethoxy-2-phenylacetophenone (DMPA) and UV light of 365 nm. Some studies with bevacizumab showed sustained drug release in vitro and in vivo; however, phototoxicity could occur [132,133]. Polyacrylic acid (PAA) and hydroxymethylcellulose solutions are liquids at pH 4.0, but are transformed into viscous gels at pH 7.4. These pH-sensitive and ion-sensitive polymers are mainly employed to develop topical eye drops in anterior segment diseases [134,135].

### 3.6. Combined Drug Delivery Systems

The combined use of micro-, nanoparticles, and hydrogels as a drug delivery system is advantageous relative to the use of each as separate systems. Micro- and nanoparticles migrate and distribute in the intraocular space and are cleared rapidly. Moreover, initial burst release occurs; hydrophilic hydrogels cause fast diffusion and short release of drugs [136]. When combined together, these disadvantages could be overcome, as hydrogels act as secondary carriers, localize nanoparticles in the injection site, and extend drug release time, thereby enhancing the drug delivery potential [136]. Liu et al. developed an injectable PNIPAAm-based thermo-responsive hydrogel with PLGA microspheres, which controlled the release of anti-VEGF drugs, such as ranibizumab or aflibercept, for up to 6 months [137,138]. In vitro studies have also revealed drug efficacy in a laser-induced CNV model. Kim et al. experimented with this aflibercept-loaded microsphere and hydrogel combination system using the nonhuman primate model, rhesus macaques. Based on their findings, effective treatment was observed until 6 months post-injection, without adverse events [139]. These combined drug delivery systems could enable fewer intravitreal injections of anti-VEGF drugs and reduce the overall treatment burden.

### 3.7. Port Delivery Systems

The port delivery system is a state-of-the-art nondegradable, refillable implant that is surgically placed in the scleral and pars plana. Passive diffusion due to concentration gradient results in movement of drugs from the port to the vitreous cavity, with sustained and controlled release achieved by the porous metal element [140]. Recently, Campochiaro et al. carried out a phase 2, multicenter, randomized, active treatment-controlled clinical trial of a ranibizumab-loaded port delivery system (LADDER; NCT02510794) to determine the efficacy and safety of the drug [140]. Participants were administered ranibizumab at 10 mg/mL, 40 mg/mL, 100 mg/mL, or monthly intravitreal ranibizumab 0.5 mg injections. After 9 months, visual outcomes were similar between patients administered the ranibizumab-loaded port 100 mg/mL and monthly intravitreal injections. Because the median time to initial refill was 15 months in the 100 mg/mL group, a significant reduction in treatment burden was expected in the port delivery system compared to intravitreal injections. Phase 3 clinical trials for neovascular AMD (ARCHWAY; NCT03677934), diabetic retinopathy (PAVILION; NCT04503551), and diabetic macular edema (PAGODA; NCT04108156) are currently in progress.

## 4. Drug Administration Routes besides the Intravitreal Route

**Figure 3:**
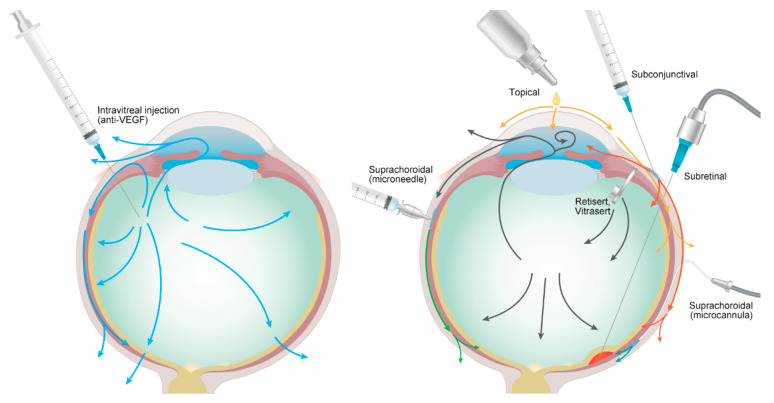
Schematic displaying intraocular distribution and elimination pathways of standard anti-VEGF intravitreal treatment and other drug administration routes. After intravitreal injection, small protein anti-VEGF antibodies move toward the anterior and posterior segment of the eye and are cleared by both pathways. Topical agents are mainly distributed in the anterior segment due to multiple physical barriers, such as the cornea and conjunctiva; however, recent drugs have utilized trans-scleral diffusion to the target posterior segment. Subconjunctival agents could also reach the posterior segment via trans-scleral diffusion of the particles. Because drug agents can move directly into the posterior segment via the suprachoroidal space, the suprachoroidal routes, via microcannula and microneedle, are promising. Subretinal injection into the subretinal space could also be employed to directly target the retinal layer.

Ocular drug delivery can be achieved via multiple routes of administration, such as the topical, systemic, intravitreal, and periocular routes. Topical administration, notably eye drops, is the ocular drug delivery route that guarantees high compliance by patients due to ease of use [188]. However, because of several anatomical and physiological barriers in the anterior segment, such as tear turnover, blinking process, and cornea and conjunctival barriers, topical formulations are inefficient via retinal drug delivery [189,190]. Systemic administration, mainly via the intravenous and oral routes, is another method of transport to the posterior segment of the eye via the choroidal capillaries. However, the blood-retinal barrier and blood-aqueous barrier block the penetration of drug molecules. Accordingly, a higher dose is needed to achieve drug efficacy, leading to increased drug toxicity [191,192]. Although oral and intravenous bioavailability is high, the systemic side effects cannot be neglected. Therefore, in ocular drug delivery systems, only few drugs, such as antibiotics, antivirals, and antineoplastic agents, have been used in clinical settings [193,194,195]. Nowadays, intravitreal drug injections are extensively used for various retinal diseases, and as presented above, intraocular pharmacokinetics and efforts to enhance the drug efficacy have been extensively investigated. Intravitreal administration has several advantages compared to other routes; this is because drugs can be directly delivered into the vitreous and retina, ultimately avoiding the barriers of the anterior segment. Relatively lower drug doses, reasonable bioavailability, and freely manipulated drug molecules could be strengths for efficient drug delivery while minimizing toxicity [196,197]. However, the direct intravitreal injection technique for puncturing the sclera is invasive, and the risks of elevated intraocular pressure, infection, and inflammation should be considered [198]. Preference for minimal invasiveness and the need to maintain efficiency has led to investigation of periocular routes. Several periocular routes, such as topical, subconjunctival, suprachoroidal, subretinal, and trans-scleral routes have been evaluated and are discussed in more detail in the following section and summarized in Table 2. Overall, Figure 3 demonstrates the standard anti-VEGF intravitreal treatment, other drug administration routes, and the intraocular distribution and elimination pathways. After intravitreal injection, anti-VEGF antibody is distributed toward the anterior and posterior segments of the eye and cleared through the aqueous, ciliary body, and retina. When other administration routes are employed, the drug agents reach the posterior segment of the eye via the suprachoroidal space, subretinal space, or trans-scleral diffusion and are eliminated by both the anterior and posterior pathways.

### 4.1. Topical Routes

The topical route is a very efficient administration route for the treatment of anterior segment diseases. However, because of multiple ocular barriers, such as tear drainage, cornea, conjunctiva, and blood-aqueous barrier, it is considered significantly inefficient for posterior segment diseases. Nevertheless, because eye drops are more convenient to administer via the topical route than others, researchers have focused on developing topical formulations to treat retinal diseases. Loftsson et al. and Sigurdsson et al. previously designed dexamethasone-cyclodextrin complexes for topical drug delivery to the posterior segments of the eye [141,142]. Recently, topical regorafenib, a multikinase inhibitor used to treat neovascular AMD, was investigated in an open-label phase 2 clinical trial (DREAM; NCT02222207) to determine its efficacy [143]. By week 12, 21 (41%) of the 51 subjects required intravitreal ranibizumab rescue due to ocular treatment-emergent adverse events. As a result, the researchers terminated the clinical trial and suggested that the trial’s failure was due to insufficient exposure of the posterior segment to the topically administered drug. Another topical agent developed by PanOptica (Mount Arlington, NJ), PAN-90806, is a tyrosine kinase inhibitor of VEGF-A and PDGF. Previously, a phase 1/2 clinical trial (NCT03479372) was performed with treatment-naïve neovascular AMD patients [144] who were administered eye drops (2, 6, and 10 mg/mL) daily for 12 weeks. Each week, the need for rescue intravitreal injections was evaluated. Based on the study findings, no rescue injections were required for 51% of the participants. Moreover, improved visual outcomes were observed in 88% of patients in the no rescue group [144]. 

### 4.2. Subconjunctival and Subtenon Routes

The subconjunctival route is a minimally invasive route for ocular drug delivery to the posterior segment. Drug injection or implants in the subconjunctival space skip the conjunctival and corneal barriers and display a higher permeability via the retina/choroidal area [145]. Accordingly, the lower probability of risks associated with intravitreal injections, such as endophthalmitis, intraocular inflammation, retinal toxicity, and retinal detachment, is thus promising with the subconjunctival routes [145]. Nonetheless, scleral barriers to the choroid and elimination in the blood and lymphatic flow in the subconjunctival space result in low retinal bioavailability [146]. In previous studies, approximately 80–90% of small molecular drugs administered in the subconjunctival space were rapidly absorbed into systemic circulation (blood and lymphatic flow) [146]. The rich and active network of lymphatic systems found in the conjunctiva plays a significant role in the drug clearance pathway [146]. Therefore, the subconjunctival route has been considered to be limited based on drug efficiency and controlled release.

Numerous experiments and studies have been performed to determine the efficacy of drugs designed to treat retinal diseases. By preparing polylactic acid (PLA) nanoparticles with budesonide, Kompella et al. observed sustained release in the intraocular space [147]. Van Quill et al. developed fibrin sealant carboplatin to treat retinoblastoma; this drug was subconjunctivally injected into mouse eyes [148]. Ayalasomayajula et al. administered celecoxib-polylactic-co-glycolic acid microparticles to treat diabetic retinopathy. Based on their in vitro experiments, sustained release of the drug occurred for 7 weeks, with a high concentration delivered to the retinal layer [149]. Misra et al. developed a subconjunctival insulin-loaded hydrogel implant to achieve sustained release in the intraocular space for diabetic retinopathy [150]. Kang et al. fabricated dendrimeric carboplatin nanoparticles for retinoblastoma. Compared to conventional treatment, the outcomes were found to be better after subconjunctival injection into mouse eyes [151]. After preparing ovalbumin with PLGA-, polyethyl glycol-degradable gel, Rieke et al. injected this formulation into rat eyes. In vitro, an initial burst release occurred followed by sustained release for up to 20 days; however, in vivo, sustained release occurred for up to 2 weeks [152]. Peng et al. produced biodegradable subconjunctival microfilm implants modified with PLGA and polycaprolactone (PCL) in rabbit eyes [153]. The presence of implants for up to 6 months, with slow degradation in the subconjunctival space, led to controlled drug release in the intraocular space. Nagai et al. developed non-degradable polymeric implants conjugated with fluorescents. Further, they reported the detection of fluorescence in the ocular tissue after 4 weeks in rats, without initial burst release [154]. Imai et al. fabricated insulin-loaded hydrogel implants that exhibited sustained release for up to 2 months in rats [155].

The subtenon route is another periocular drug administration route that increases delivery to the posterior segment of the eye. Reduced elimination via systemic circulation and the prolongation of scleral contact duration have enabled promising therapeutic effects in various retinal diseases, especially subtenon triamcinolone injection into patients with diabetic macular edema and posterior uveitis [156]. After minimal surgical incision of the Tenon’s capsule, a blunt-tipped cannula needle is often placed at the subtenon space. A relatively large volume of up to 4 mL behind the equator around the muscle belly results in slower clearance and facilitation of trans-scleral drug penetration into the vitreous [157]. However, elimination via choroidal circulation causes reduced drug efficacy [157].

### 4.3. Suprachoroidal Routes

Suprachoroidal administration is another minimally invasive technique for drug delivery to the posterior segment of the eye. After the drug is delivered to the suprachoroidal space via this pathway, it can directly target retinal layers and the choroid due to the posterior pole fluid flow, thereby avoiding multiple ocular tissue barriers and accomplishing drug efficacy at low dose concentrations [158,159,160]. In addition, sustained release could be achieved due to drug accumulation and distribution in the suprachoroidal area [160]. However, the risks of hemorrhage and choroidal detachment could not be neglected. Moreover, applying suprachoroidal injection by microneedles and microcannulation has disadvantages; the needle lengths and pressure must be optimized to enable sufficient drug delivery into the suprachoroidal space [161,162].

Numerous studies and experiments on the suprachoroidal routes have been conducted. In fact, early preclinical studies used catheter insertion to deliver bevacizumab through a surgical incision [163,164]. Patel et al. developed a minimally invasive injection technique with suspensions containing micro- and nanoparticles [165,166]. Further, Rizzo et al. produced a microcatheter to deliver bevacizumab into the suprachoroidal space to treat macular edema. As a result, hard exudates and macular edema were resolved [167]. Edelhauser et al. and Chen et al. prepared a suprachoroidal injection of triamcinolone acetonide for delivery into rabbit eyes. As a result, a greater drug efficacy was achieved with higher concentrations in the retina/choroid than that achieved on subtenon and intravitreal administration [168,169].

Human clinical trials with suprachoroidal injections are currently in progress. A phase 2 study (TANZANITE; NCT02303184), which assessed suprachoroidal investigational triamcinolone acetonide formulation (CLS-TA) in combination with intravitreal aflibercept injection for the treatment of retinal vein occlusion with macular edema, was performed by Campochiaro et al. [170]. Functional and anatomical visual outcomes were significantly improved relative to those achieved with aflibercept monotherapy. Further, less frequent aflibercept intravitreal injections were noted. However, a phase 3 trial suggested that there are no benefits to combined suprachoroidal treatment. Wykoff et al. evaluated intravitreal aflibercept treatment with or without suprachoroidal CLS-TA injection for diabetic macular edema; however, a phase 2 trial (TYBEE; NCT03126786) revealed no definite advantages in the visual outcomes with combined therapy [171]. A phase 3 masked, randomized trial of the suprachoroidal administration of CLS-TA with microneedles for noninfectious uveitis complicated by macular edema (PEACHTREE; NCT02595398) was performed by Yeh et al. [172] CLS-TA was injected on day 0 and in week 12. Based on the results, at week 24 (the end point), the best-corrected visual acuities were improved by 15 or more ETDRS letters (47% CLS-TA vs. 16% sham, *p* < 0.001), and the thickness of the central subfield was significantly reduced (153 μm CLS-TA vs. 18 μm sham, *p* < 0.001). No serious adverse events related to suprachoroidal injection were reported, and a lower probability of elevated intraocular pressure and cataract was calculated.

Although there are no commercialized drug delivery devices for the suprachoroidal route, new minimally invasive treatment methods for various retinal diseases are expected from ongoing animal studies and human clinical trials.

### 4.4. Subretinal Routes

The subretinal space is located between the RPE layer and the photoreceptors. Under a surgical microscope, drug administration can be performed with direct visualization [173]. The trans-corneal or trans-scleral route has mainly been employed in animal experiments to derive subretinal injection techniques [174]. Subretinal delivery is very useful as it enables direct drug effects on subretinal space cells and the retinal layer. Recently, subretinal injections have been used to treat inherited retinal diseases, ultimately delivering genes directly to the RPE and photoreceptor cells. Johnson et al. injected DNA-carried viral vectors into the subretinal space to evaluate this gene therapy for pigmentary retinitis and Leber’s congenital amaurosis [175]. Rajala et al. administered liposome-assisted RPE65-related DNA to achieve direct DNA delivery to RPE cells and to gain long-term gene expression in vivo [176]. Sun et al. developed dendrimer-assisted plasmid DNA to determine the efficiency of subretinal delivery [177]. Further, Apaolaza et al. used nanoparticle-based vectors to treat X-linked juvenile retinoschisis in an Rs1h-deficient mouse model. Based on their findings, the delivered retinoschisin maintained its expression in the retinal layer for at least 2 months. Further, partial recovery of the retina was observed in vivo [178].

RGX-314, an AAV8 vector that delivers genome to induce anti-VEGF Fab antibody production, is a notable ongoing human clinical trial on subretinal administration. Previously, Regenxbio performed a Phase 1/2a, open-labeled, nonrandomized (NCT03066258) trial. After the initial ranibizumab injection, genome copies were injected and an anti-VEGF was subsequently administered after 4 weeks, as needed [179]. Based on the study findings, no adverse events occurred until 6 months, and visual outcomes were significantly improved, with less intravitreal injections required relative to standard treatment; this human trial continued to the secondary endpoints at week 106.

### 4.5. Trans-Scleral Routes

Trans-scleral iontophoresis is a representative noninvasive drug administration device for delivering macromolecules to the posterior pole of the eye. The mechanisms of ocular iontophoresis include electrophoresis, electroosmosis, and electro-permeabilization with a low electric current applied to the sclera [180,181]. Previous studies focused on the delivery of micromolecules, such as carboplatin, methotrexate, and methylprednisolone, to the posterior segment of the eye [180,181,182,183,184,185,186,187]. In an experimental uveitis model using rabbit model, Papangkorn et al. applied Visulex-P (developed by Aciont Inc., Salt Lake City, UT, USA), which employs the trans-scleral route with passive diffusion for drug delivery [180]. Compared to intravitreal injections, the device successfully suppressed uveitis inflammation in a month. Recent studies have shown that macromolecules up to 150 kDa (molecular weight) can also be transported via the sclera. In fact, bevacizumab was previously demonstrated to be a promising drug for delivery through the human sclera with anodal iontophoresis [182]. The latest study by Molokhia et al. suggested that macromolecules, including immunoglobulin G (IgG) and bevacizumab, could be transported with transscleral iontophoresis in vitro and in vivo. Further, in rabbit CNV model, delayed retinal neovascularization was observed in week 8, with similar treatment effects to conventional intravitreal injections but without serious adverse events [183]. Further studies with anti-VEGF drugs should thus be conducted to derive its potential for clinical application.

## 5. Conclusions and Future Perspectives

**Figure 4:**
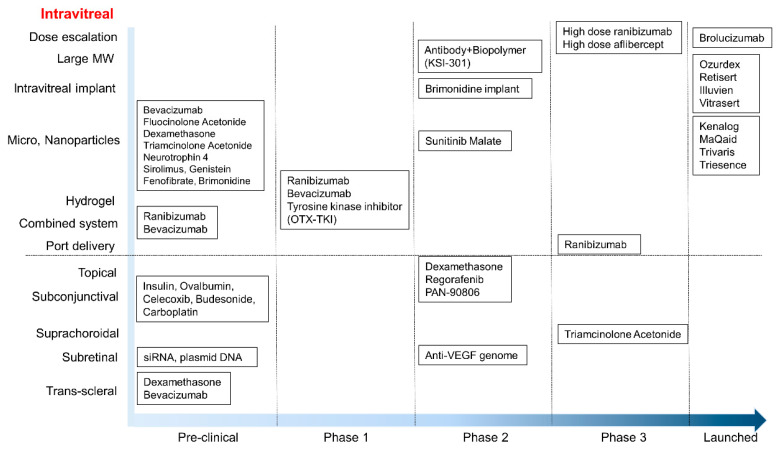
Summary of current development and human clinical trials for enhanced ocular drug delivery to the posterior segment of the eye.

Over the past decade, ocular drug delivery to the retina has been extensively investigated. In fact, after the development of anti-VEGF drugs, the commercial market has markedly expanded. Representative anti-VEGF drugs, including bevacizumab, ranibizumab, aflibercept, and the recently added, brolucizumab, target the posterior segment of the eye for retinal neovascularization. Although the treatment effect of these drugs has saved patients from devastating vision loss, frequent intravitreal injections and their accompanying cost burden have highlighted the need for advanced minimally invasive techniques and sustained drug release methods. To achieve sustained drug release, dose escalation, conjugation of micro-and nanoparticles, hydrogels, and combined systems have thus been investigated. For minimally invasive techniques, periocular administration, such as subconjunctival, suprachorodial, subretinal, and trans-scleral injections, has been investigated in human clinical trials. Figure 4 and Table 3 summarizes current development and human clinical trials that aim to enhance ocular drug delivery to the posterior segment of the eye.

Because of the increase in the number of patients with vision-threatening retinal diseases, such as AMD, and several indications of anti-VEGF agents in real-world ophthalmic practice, the demand for ophthalmic biologic drugs is increasing. Ranibizumab [199] and aflibercept biosimilars, the latest biosimilar anti-VEGF drugs, are currently in human clinical trials. Compared to the approved reference biological agents, biosimilar products do not display any clinically meaningful differences in their pharmacokinetic and pharmacodynamic properties and present a markedly lower cost burden on patients. The use of biologics for posterior ocular drug delivery will increase due to increased competition between original biologic drugs and biosimilars; further, there will be fewer issues of patents. However, challenges still remain when biologic drugs are employed in various ocular drug delivery systems, including the maintenance of their three-dimensional structures and drug efficacy. During loading, protein drugs can be denatured into degradable implants and particles, such as PLGA implants. Although hydrogel systems can deliver protein drugs and maintain their efficacy, loading a sufficient dose of protein drugs in a limited volume of hydrogel systems remains a challenge. The ability to develop novel ophthalmic drug delivery methods carrying biologic drugs into the posterior part of the eyeballs will thus be a fiercely competitive challenge in the future.

Future ideal ocular delivery systems should be developed to meet the requirements of sustained drug release and minimally invasive administration and should maintain the efficacy of the drug in the intraocular space. Advanced nanotechnology and tissue engineering should be employed to develop long-acting drug molecules with high ocular biocompatibility. Accordingly, no definite serious local and systemic adverse effects should occur. Moreover, modified molecular sizes and surfaces and specific retinal cell targeting (RPE and photoreceptors) are needed to improve drug efficacy. In addition to the performance of consistent human clinical trials after pre-clinical studies, detailed and long-term investigations on ocular toxicity and biocompatibility should be performed, with focus on the biochemical, immunological, and ophthalmological aspects.

Recently, outbreaks of intraocular inflammation with obstructive retinal vasculitis were reported after brolucizumab was administered via the intravitreal route. Patients and physicians are thus highly wary of this complication [200,201,202]. These inflammatory adverse events, which could be caused by any intraocular drug, will also be carefully monitored by the regulatory authorities. Moreover, new ocular drug delivery systems should be developed for entry into the market and clinic. Similar to new intraocular drugs, future intraocular drug delivery systems should be carefully tested during the developmental stage to determine their potential to induce intraocular inflammation.

## Figures and Tables

**Table 2:** Ocular drug delivery systems to the retina.

Route	Delivery Platform		Characteristics	Drug/Cargo	Development	Reference
Intravitreal	Implants		Biodegradable and non-biodegradable implants Prolonged intravitreal half-lives and easy to administer Biodegradables require 3–6 months repeated injections Non-biodegradables are retained in the vitreous cavity	Dexamethasone(Ozurdex) Fluocinolone acetonide (Retiset, Illuvien) Ganciclovir (Vitrasert) Brimonidine (Brimo DDS)	Launched Phase 2	[62,63,64,65]
	Micro-, Nano-particles	Microparticles	Microcrystal particles change to depot formulation in the vitreous cavity Good bioavailability, slow release, long-lasting therapeutic effect	Triamcinolone (Kenalog, MaQaid, Trivaris, Triesence) Sunitinib Malate (GB-102)	Launched Phase 2	[70,71,72,73,74,75,76,77,78]
		Dendrimer	3D repeating-unit branched macromolecules PAMAM and PPI/DAB Encapsulated therapeutics via hydrogen bonds, hydrophobic interactions, or ionic interactions	Anti-VEGF (Bevacizumab) Fluocinolone Acetonide Dexamethasone Triamcinolone Acetonide Neurotrophin 4	Pre-clinical	[79,80,81,82,83,84,85]
		Liposome	Vesicle composed as a single or multiple bilayered lipidic membranes and an aqueous core Capable of encapsulating diverse physicochemical Cproperties therapeutics Low toxicity and good bioavailability	siRNA DNA Anti-VEGF (Bevacizumab)	Pre-clinical	[86,87,88,89,90]
		Micelles	Core−shell structures of amphiphilic copolymers PEG/PEO (hydrophilic shell) PGA/PCL/PLA/PLGA (hydrophobic core) Capable of encapsulating diverse physicochemical properties therapeutics Good drug solubility, increased retention	Dexamethasone Triamcinolone Acetonide Sirolimus Genistein	Pre-clinical	[91,92,93,94,95,96,97]
		Polymeric	Encapsulated or dispersed therapeutics in the polymer matrix (PGA, PLA, PLGA with PVA, PVP) Good bioavailability, increased retention	Anti-VEGF (Bevacizumab) Fenofibrate	Pre-clinical	[98,99,100,101]
		Others	Solid lipid Human serum albumin, chitosan, cyclodextrin Inorganic	Anti-VEGF (Bevacizumab) Brimonidine	Pre-clinical	[102,103,104,105,106,107,108,109,110,111,112,113,114,115,116,117,118]
	Hydrogel		Multi-dimensional network structure with cross-linked polymer chains In situ hydrogels with sol-gel transformation by external stimuli (temperature, light, pH, ion)	Anti-VEGF (Ranibizumab, Bevacizumab) Tyrosine kinase inhibitor	Phase 1 (Ocular therapeutix)	[119,120,121,122,123,124,125,126,127,128,129,130,131,132,133,134,135]
	Combined systems		Nanoparticles + Hydrogels Long retention time, less treatment burden	Anti-VEGF (Ranibizumab, Bevacizumab)	Pre-clinical	[136,137,138,139]
	Port delivery system		Surgically inserted permanent, refillable port Long retention time, less treatment burden	Anti-VEGF (Ranibizumab)	Phase 3	[140]
Topical			Easy to administer, good drug compliance Very inefficient to reach posterior segment	Dexamethasone Regorafenib PAN-90806	Phase 2	[141,142,143,144]
Subconjunctival	Hydrogel		Skip conjunctival and corneal barriers, and provide higher permeability through retina/choroidal area Much less invasive than intravitreal injections	Insulin Ovalbumin	Pre-clinical	[145,146,147,148,149,150,151,152,153,154,155,156,157]
	Polymeric Nanoparticles			Budesonide Carboplatin Celecoxib	Pre-clinical	
Suprachoroidal	Microneedle Microcannulation		Directly target retinal layers and choroid Reach drug efficacy with low dose concentration Possibility of hemorrhage and choroidal detachment Needle length and pressure should be optimized	Triamcinolone Acetonide	Phase 3	[158,159,160,161,162,163,164,165,166,167,168,169,170,171,172]
Subretinal	Liposome Dendrimer Viral vector		Direct drug effect on subretinal space and retinal layer Possible route for gene therapy	siRNA Plasmid DNA Anti-VEGF genome	Pre-clinical Phase 1/2	[173,174,175,176,177,178,179]
Trans-scleral	Iontophoresis		Electrophoresis, Electroosmosis Electro-permeabilization	Dexamethasone Anti-VEGF (Bevacizumab)	Pre-clinical	[180,181,182,183,184,185,186,187]

**Table 3:** Previous and current human clinical trials on the advancements made regarding ocular drug delivery systems.

Drug Delivery Systems	Trial Name	Drug (Sponsor)	Description	Results
Dose escalation	HARBOR (NCT00891735)	Ranibizumab (Novartis)	Phase 3, interventional, multicenter, randomized, dose-comparison	2.0 mg (4-fold dose escalation) showed similar treatment effect compared to 0.5 mg dose, whereas fewer required injections.
	SAVE (NCT01025232)	Ranibizumab (Novartis)	Phase 1/2a, interventional, open-label, multicenter	2.0 mg (4-fold dose escalation) showed visual and anatomic gains in recalcitrant neovascular AMD.
	READ 3 (NCT01077401)	Ranibizumab (Novartis)	Phase 3, interventional, multicenter, randomized, controlled, double-masked	2.0 mg (4-fold dose escalation) showed no superior 24 months visual improvements compared to 0.5 mg dose in diabetic macular edema.
	PHOTON (NCT04429503)	Aflibercept (Bayer)	Phase 3, interventional, multicenter, randomized, controlled, double-masked	Ongoing 8.0 mg (4-fold dose escalation) every 12 or 16 weeks after a loading phase in participants with diabetic retinopathy.
	PULSAR (NCT04423718)	Aflibercept (Bayer)		Ongoing 8.0 mg (4-fold dose escalation) every 12 or 16 weeks after a loading phase in participants with neovascular AMD.
	HAWK (NCT02307682)	Brolucizumab (Novartis)	Phase 3, interventional, multicenter, randomized, active-controlled, double-masked	Brolucizumab 3 mg showed similar visual outcomes compared to aflibercept and resulted in fewer injections.
	HARRIER (NCT02434328)	Brolucizumab (Novartis)		Brolucizumab 3 mg and 6 mg showed similar visual outcomes compared to aflibercept and resulted in fewer injections.
Large molecule biopolymer	DAZZLE (NCT04049266)	KSI-301 (KODIAK)	Phase 2b/3, interventional, multicenter, randomized, controlled, double-masked	Ongoing//KSI-301 5 mg vs. Aflibercept 2 mg Prior phase 1 study (NCT03790852) resulted in visual improvements with 6-month interval intravitreal injections in neovascular AMD, diabetic macular edema and retinal vein occlusion subjects.
Intravitreal implant	(NCT00658619)	Brimonidine (Allergan)	Phase 2, interventional, randomized, multicenter	Mean area of geographic atrophy secondary to neovascular AMD was reduced at month 12 in brimonidine implant compared to sham.
Microparticles	ADAGIO (NCT03249740)	Sunitinib Malate (GB-102, GrayBug vision)	Phase 1/2a, interventional, randomized, multicenter	88% patients at 3 months and 68% patients at 6 months were maintained on a single intravitreal injection.
	ALTISSIMO (NCT03953079)		Phase 2b, interventional, randomized, multicenter	Ongoing Compare the visual outcome after intravitreal administration of 1 mg and 2 mg GB-102, and aflibercept 2 mg dose in neovascular AMD.
	(NCT04085341)		Phase 2a, interventional, randomized, multicenter	Ongoing Diabetic macular edema and retinal vein occlusion.
Hydrogel	CLN-0046 (NCT03630315)	OTX-TKI (Ocular Therapeutix)	Phase 1, interventional, open-label, randomized, controlled, multicenter	Ongoing Low dose vs. middle dose vs. high dose vs. OTX-TKI + Anti-VEGF.
Port delivery system	LADDER (NCT02510794)	Ranibizumab (Novartis)	Phase 2, interventional, randomized, controlled, multicenter	Visual outcomes were similar in the ranibizumab-loaded port 100 mg/mL and monthly intravitreal injections at 9 months with the median time to initial refill was 15 months in the 100 mg/mL group.
	ARCHWAY (NCT03677934)	Ranibizumab (Novartis)	Phase 3, interventional, randomized, visual assessor-masked, active-comparator study	Ongoing Port delivery system 100 mg/mL vs. monthly intravitreal injection 0.5 mg (10 mg/mL) in neovascular AMD.
	PAVILION (NCT04503551)	Ranibizumab (Novartis)		Ongoing Diabetic retinopathy.
	PAGODA (NCT04108156)	Ranibizumab (Novartis)		Ongoing Diabetic macular edema.
Topical	DREAM (NCT02222207)	Regorafenib (Bayer)	Phase 2a/2b, interventional, randomized, multicenter	Among 51 subjects, 21 patients (41%) required intravitreal ranibizumab rescue due to the ocular treatment-emergent adverse events by week 12.
	(NCT03479372)	PAN-90806 (PanOptica)	Phase 1/2a, interventional, randomized, uncontrolled, double-masked, multicenter	Two, 6, or 10 mg/mL eye drops daily for 12 weeks No rescue therapy for 51% of patients. Eighty-eight percent of nonrescued patients showed clinical improvement or stability.
Suprachoroidal	TANZANITE (NCT02303184)	Triamcinolone Acetonide (Clearside Biomedical)	Phase 2, interventional, randomized, controlled, masked	Improved visual outcomes than aflibercept monotherapy. Lesser number of intravitreal injections.
	TYBEE (NCT03126786)		Phase 2, interventional, randomized, controlled, double-masked	No definite advantages of visual outcomes in diabetic macular edema with suprachoroidal CLS-TA + intravitreal aflibercept combined.
	PEACHTREE (NCT02595398)		Phase 3, interventional, randomized, controlled, double-masked	Better visual outcome and less ocular complication in noninfectious uveitis complicated by macular edema.
Subretinal	(NCT03066258)	RGX-314 (Regenxbio)	Phase 1/2a, interventional, open-label, non-randomized, multiple-cohort, dose-escalation	Ongoing After initial ranibizumab injection, genomes were injected and anti-VEGF was treated after 4 weeks and as needed. At 6 months, visual outcomes were improved with less required intravitreal injections.

## Data Availability

Not applicable.
